# Supplementary material for: A stochastic simulation-based approach to inform the relapsing mouse model study design for non-clinical assessment of tuberculosis
Source: Antimicrob Agents Chemother. 2025 Dec 29;70(2):e01103-25. doi: 10.1128/aac.01103-25 (PMC12888889; doi:10.1128/aac.01103-25)
Supplement: Supplemental material — Table S1; Fig. S1 to S16. [file aac.01103-25-s0001.docx]

**Supplementary Materials**

**STABLE 1: Comparison of true and model-estimated regimen T_95_ rank order in ascending order – Median (5^th^-95^th^ percentiles) from Simulation Round 2**

| **Regimen** | **“True” Rank** | **Original Design** | **Proposed Design 5** | **Proposed Design 6** |
| --- | --- | --- | --- | --- |
| Regimen 1 | 1 | 1  (1, 3) | 1 (1, 3) | 1 (1, 4) |
| Regimen 11 | 2 | 3  (2, 6) | 3 (1, 6) | 3 (2, 6) |
| BPaMZ | 3 | 4  (2, 5) | 4 (2, 5) | 4 (2, 6) |
| Regimen 5 | 4 | 7 (4, 9) | 7 (4, 9) | 6 (4, 9) |
| Regimen 10 | 5 | 7 (6, 10) | 7 (5, 9) | 7 (5, 9) |
| Regimen 8 | 6 | 3 (1, 6) | 3 (1, 6) | 3 (1, 7) |
| Regimen 6 | 7 | 7 (5, 10) | 7 (4, 10) | 7 (4, 10) |
| Regimen 9 | 8 | 3 (1, 7) | 3 (1, 7) | 4 (1, 8) |
| Regimen 4 | 9/10 | 10 (7, 12) | 10 (7, 12) | 10 (8, 11) |
| Regimen 12 |  | 10 (8, 12) | 10 (8, 12) | 10 (8, 11) |
| Regimen 7 | 11 | 10 (7, 11) | 9 (7, 11) | 10 (7, 11) |
| Regimen 3 | 12/13 | 12 (11, 13) | 13 (11, 13) | 12 (11, 13) |
| Regimen 2 |  | 12 (11, 13) | 12 (10, 13) | 12 (11, 13) |
| HRZE | 14 | 14 (14, 14) | 14 (14, 14) | 14 (14, 14) |

**
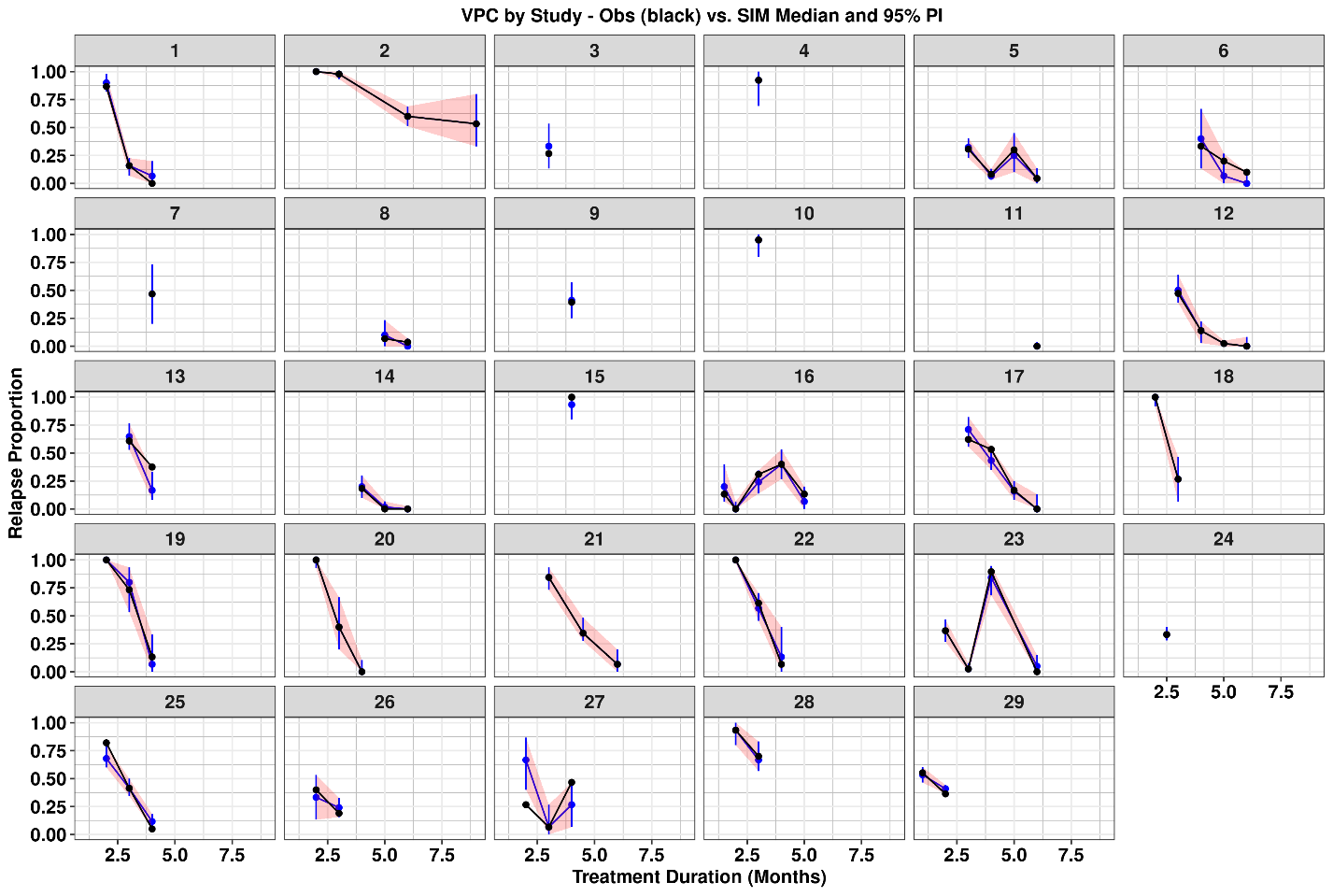
**

**SFIG1.** Visual predictive check (VPC) stratified by Study ID number (i.e., across all regimens in each study) for the model used in Round 1 simulations. Red shaded regions and blue error bars represent the 90% Prediction Interval for the model. Black points and black lines represent the actual data.


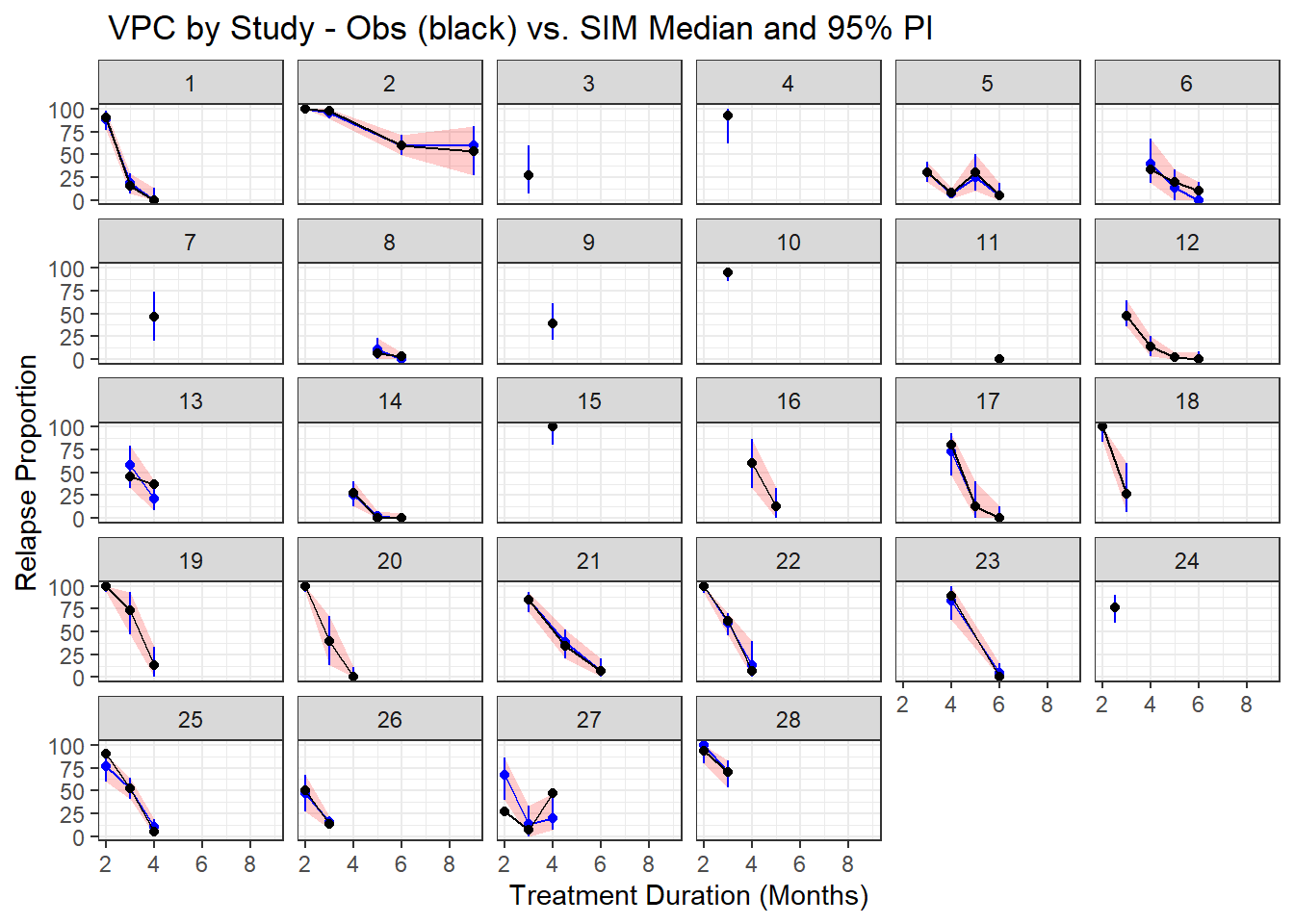
**SFIG2.** Visual predictive check (VPC) stratified by Study ID number (i.e., across all regimens in each study) for the model used in Round 2 simulations. Red shaded regions and blue error bars represent the 90% Prediction Interval for the model. Black points and black lines represent the actual data.

**
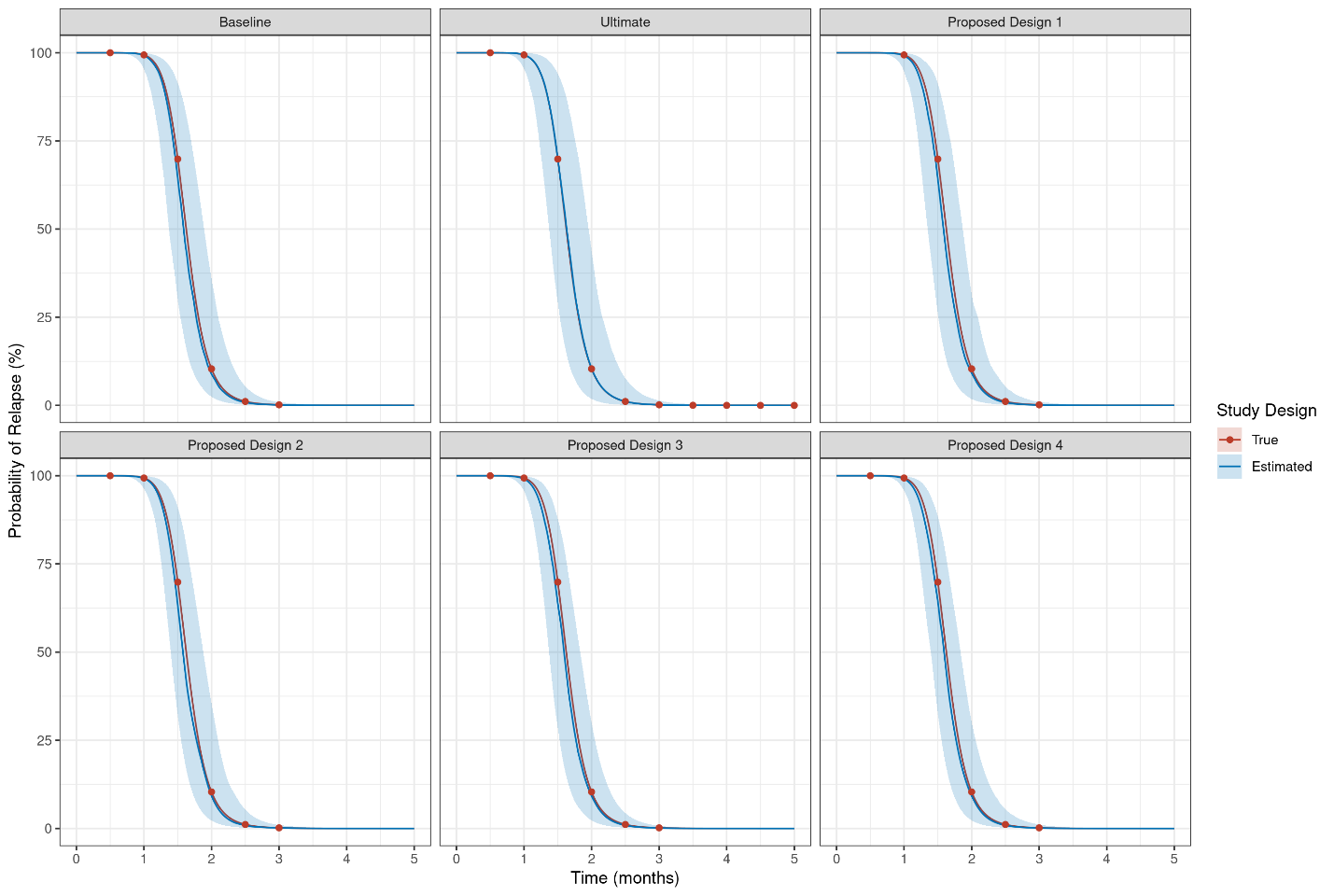
SFIG 3.** Relapse versus time profile for simulations of BPaMZ by Design for simulation round 1. Blue lines and areas represent median and 90% confidence intervals for simulations. Red lines and dots are the simulation input.

**
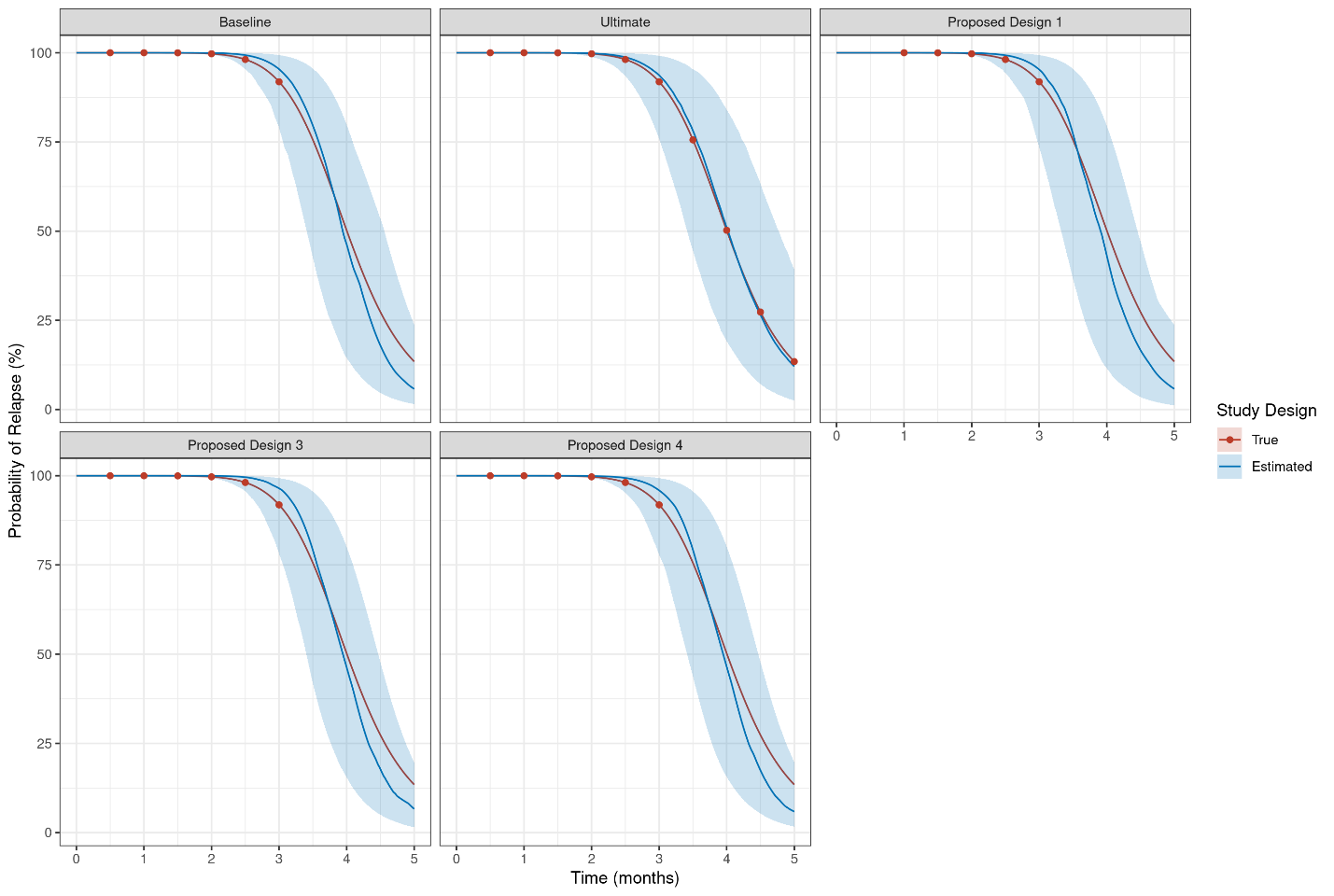
**

**SFIG 4.** Relapse versus time profile for simulations of HRZE by Design for simulation round 1. Blue lines and areas represent median and 90% confidence intervals for simulations. Red lines and dots are the simulation input.


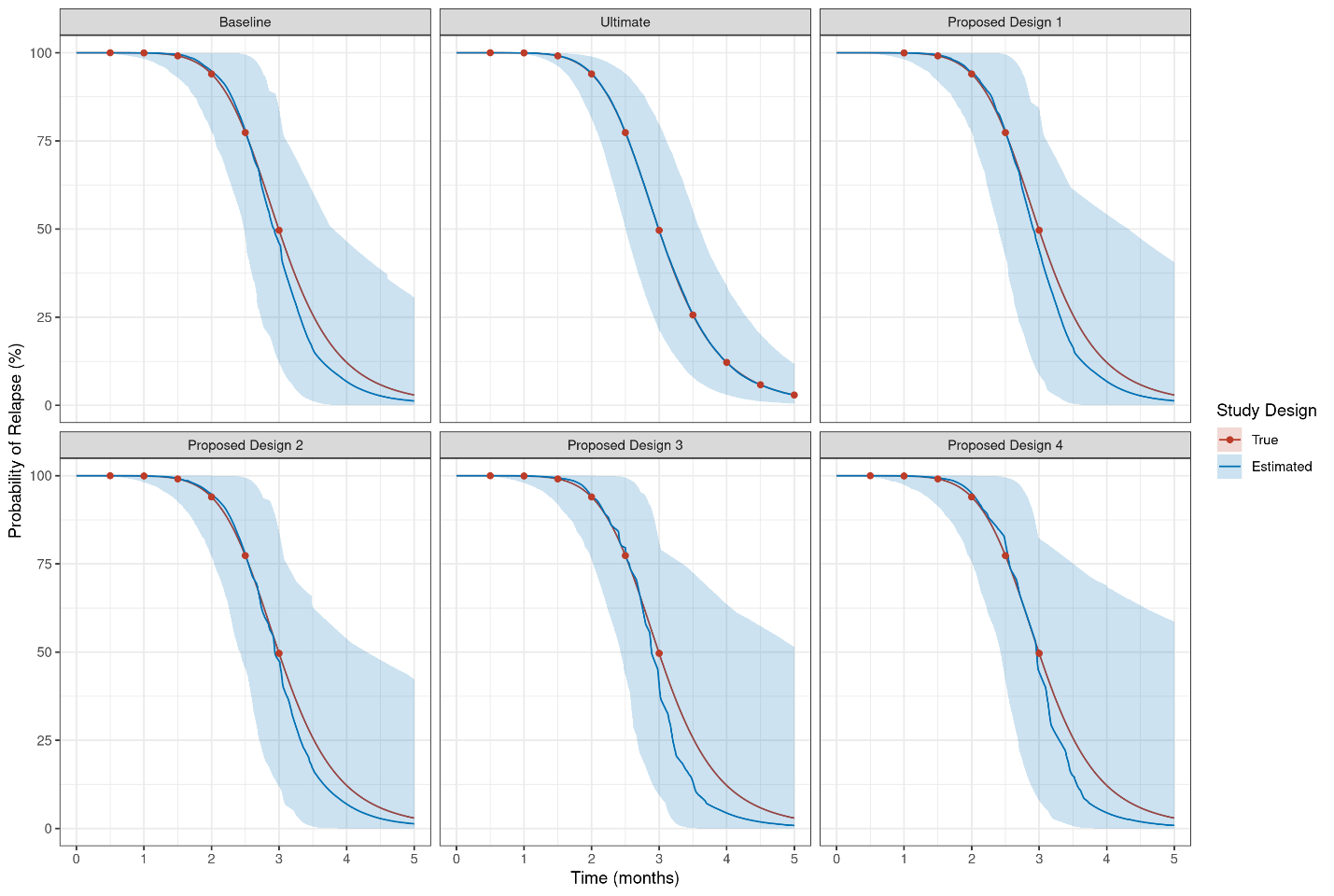
 **SFIG 5.** Relapse versus time profile for simulations of Regimen 1 by Design for simulation round 1. Blue lines and areas represent median and 90% confidence intervals for simulations. Red lines and dots are the simulation input.


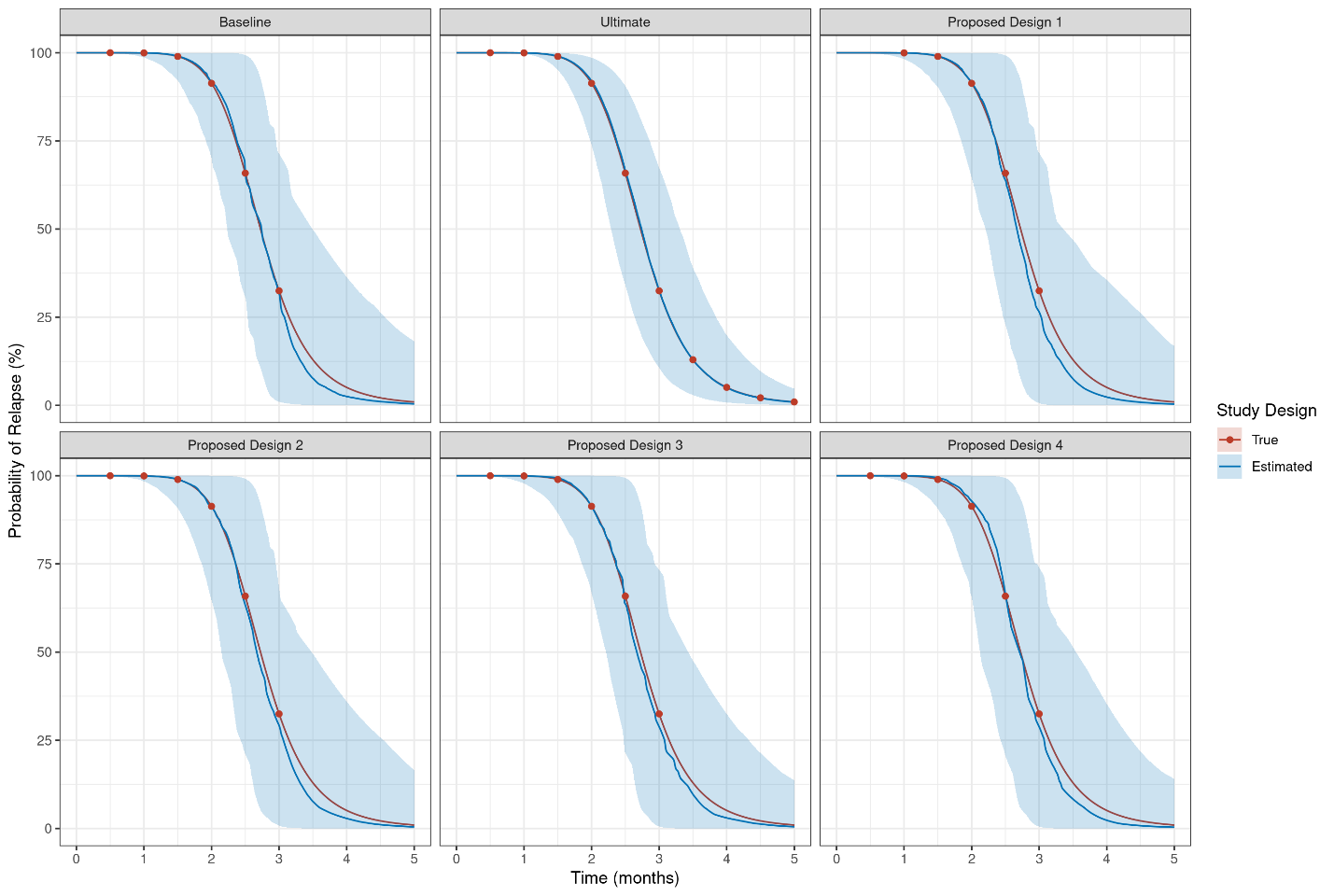


**SFIG 6.** Relapse versus time profile for simulations of Regimen 2 by Design for simulation round 1. Blue lines and areas represent median and 90% confidence intervals for simulations. Red lines and dots are the simulation input.

**
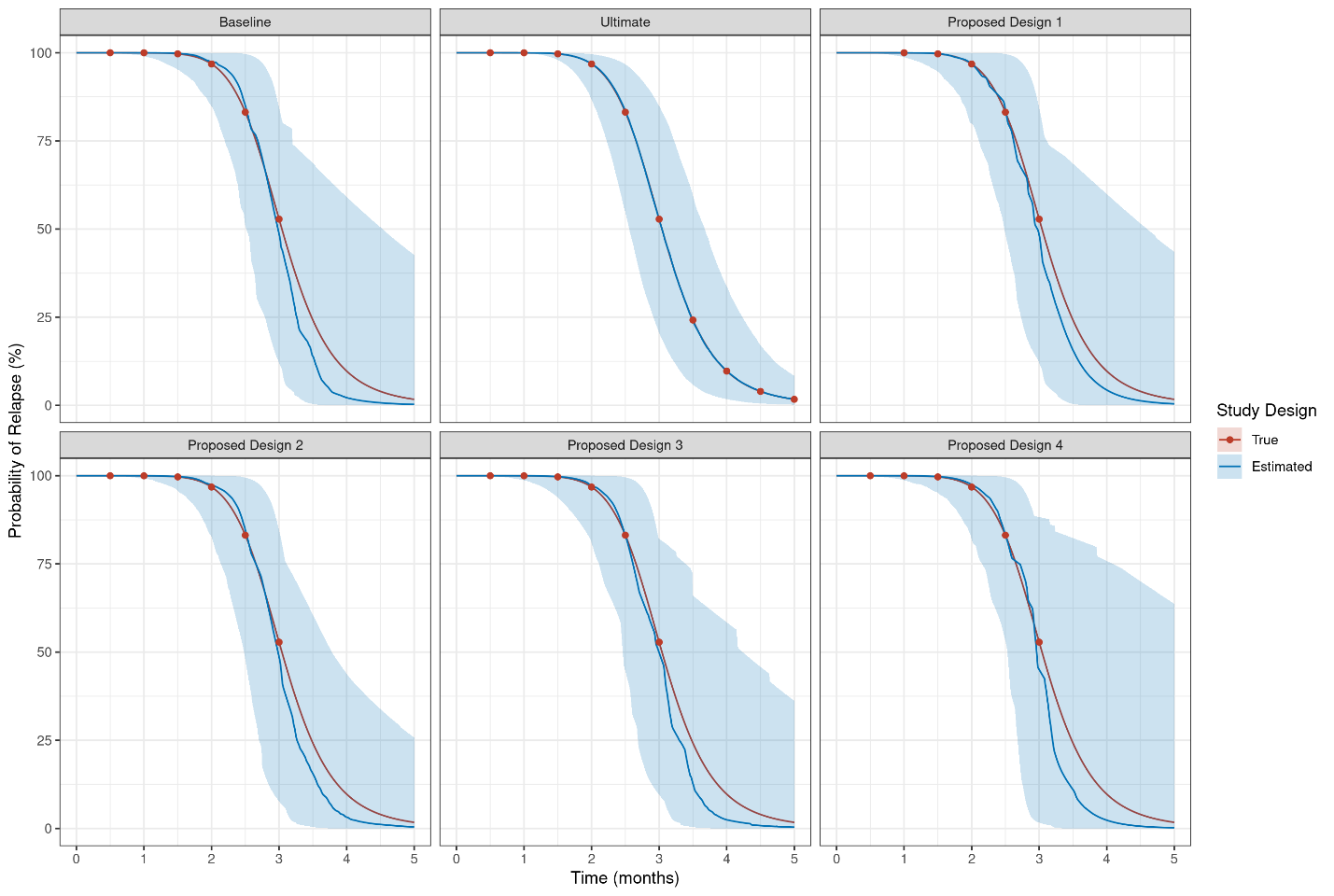
 SFIG 7.** Relapse versus time profile for simulations of Regimen 3 by Design for simulation round 1. Blue lines and areas represent median and 90% confidence intervals for simulations. Red lines and dots are the simulation input.


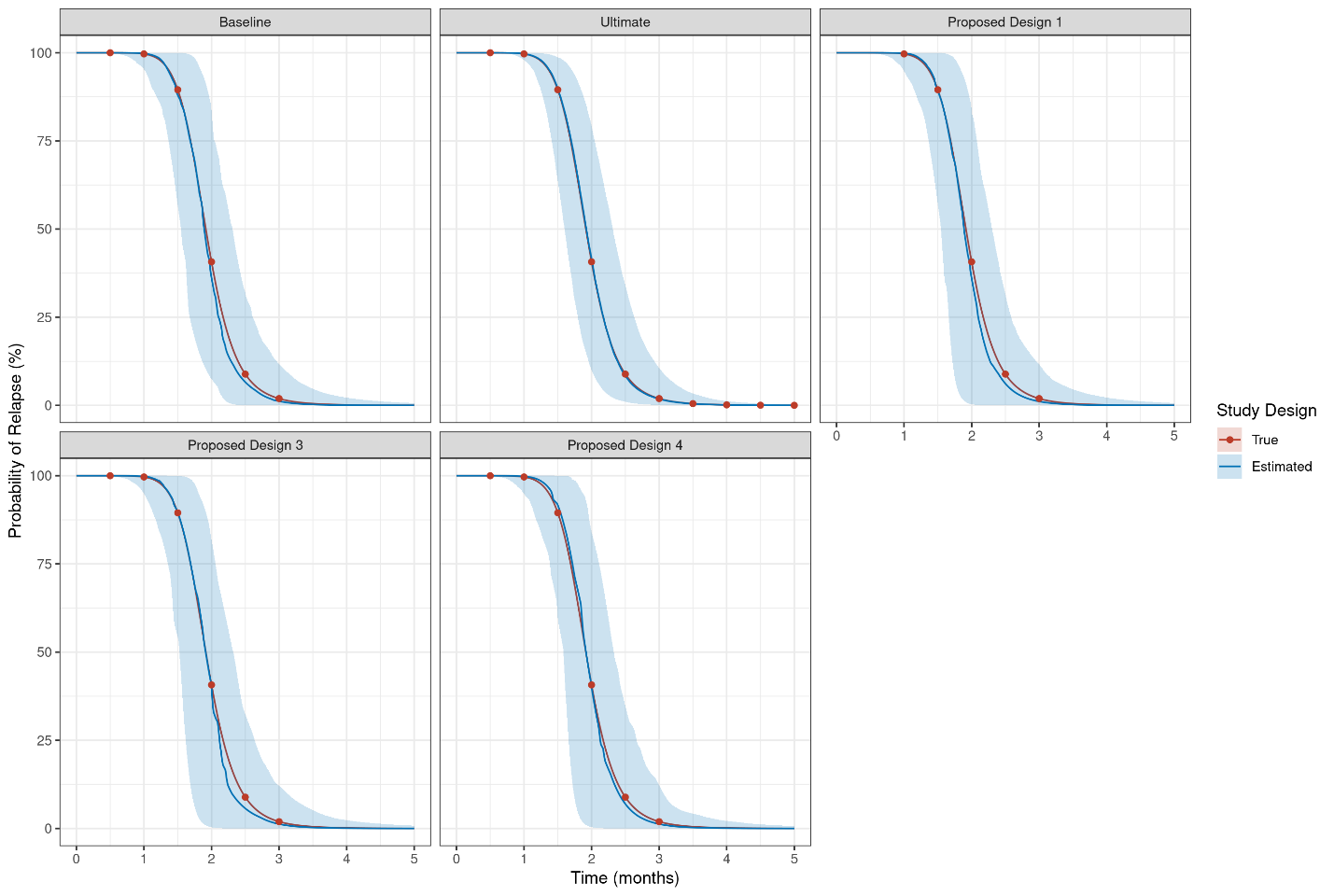
 **SFIG 8.** Relapse versus time profile for simulations of Regimen 4 by Design for simulation round 1. Blue lines and areas represent median and 90% confidence intervals for simulations. Red lines and dots are the simulation input.


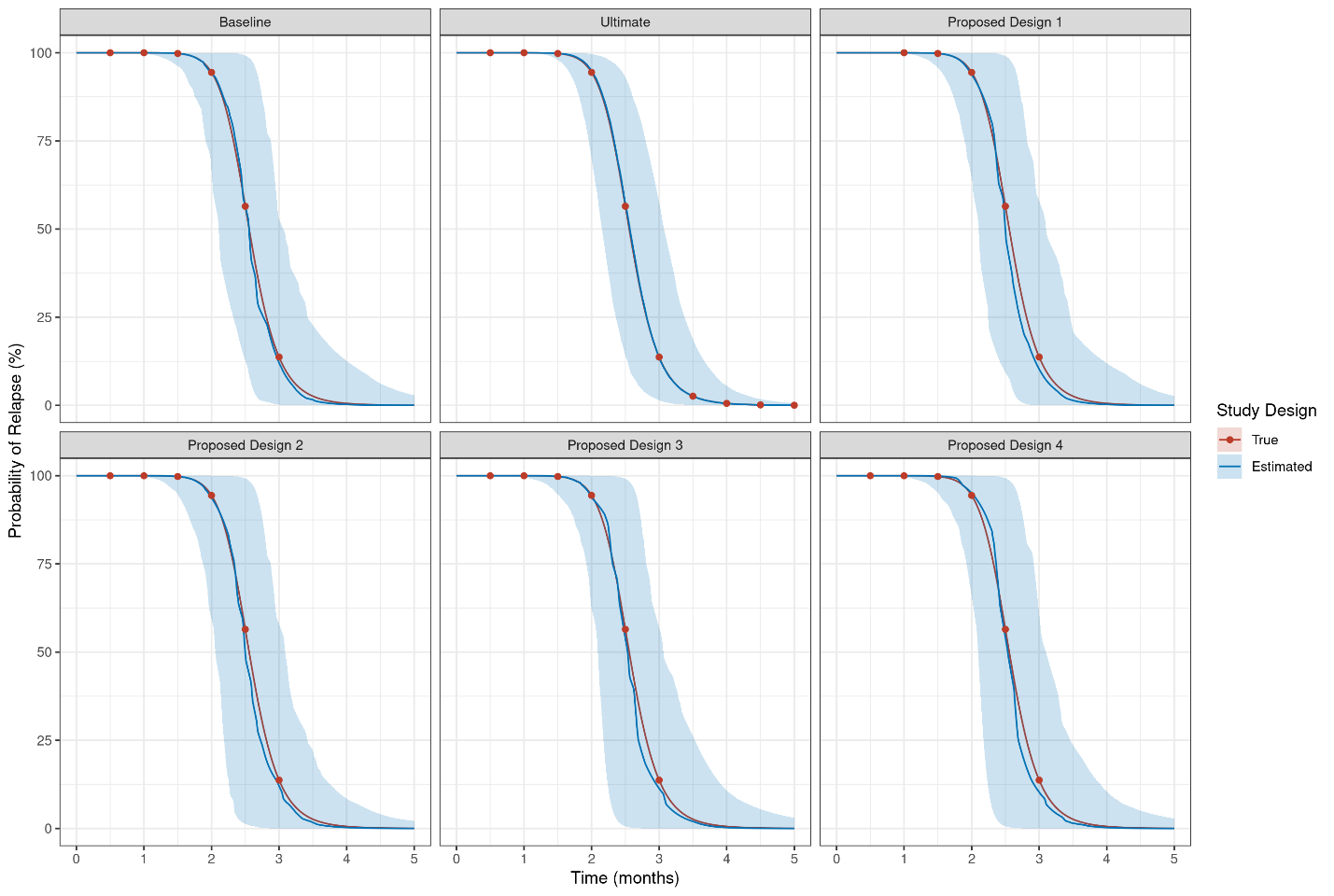
 **SFIG 9.** Relapse versus time profile for simulations of Regimen 5 by Design for simulation round 1. Blue lines and areas represent median and 90% confidence intervals for simulations. Red lines and dots are the simulation input.


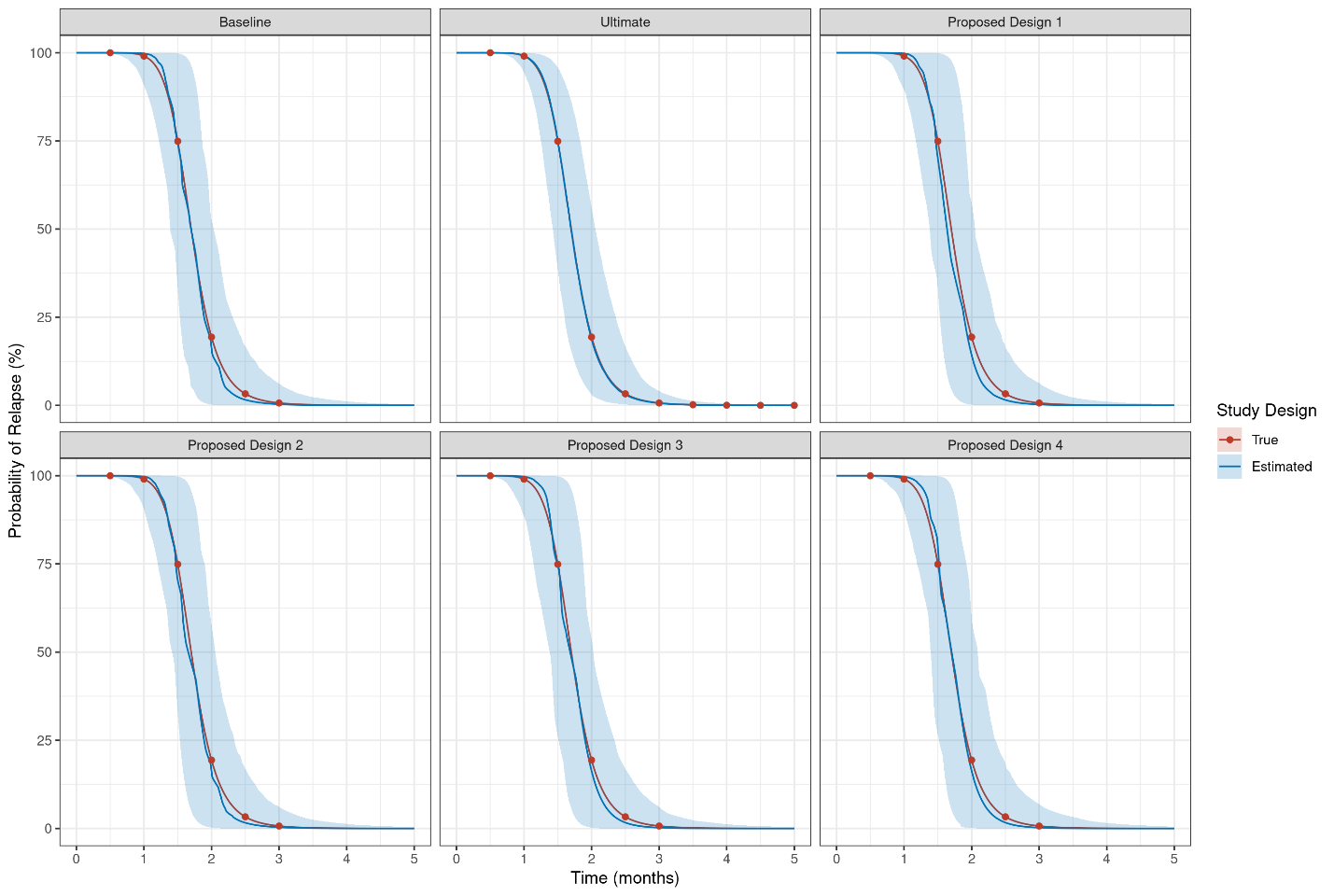
 **SFIG 10.** Relapse versus time profile for simulations of Regimen 6 by Design for simulation round 1. Blue lines and areas represent median and 90% confidence intervals for simulations. Red lines and dots are the simulation input.


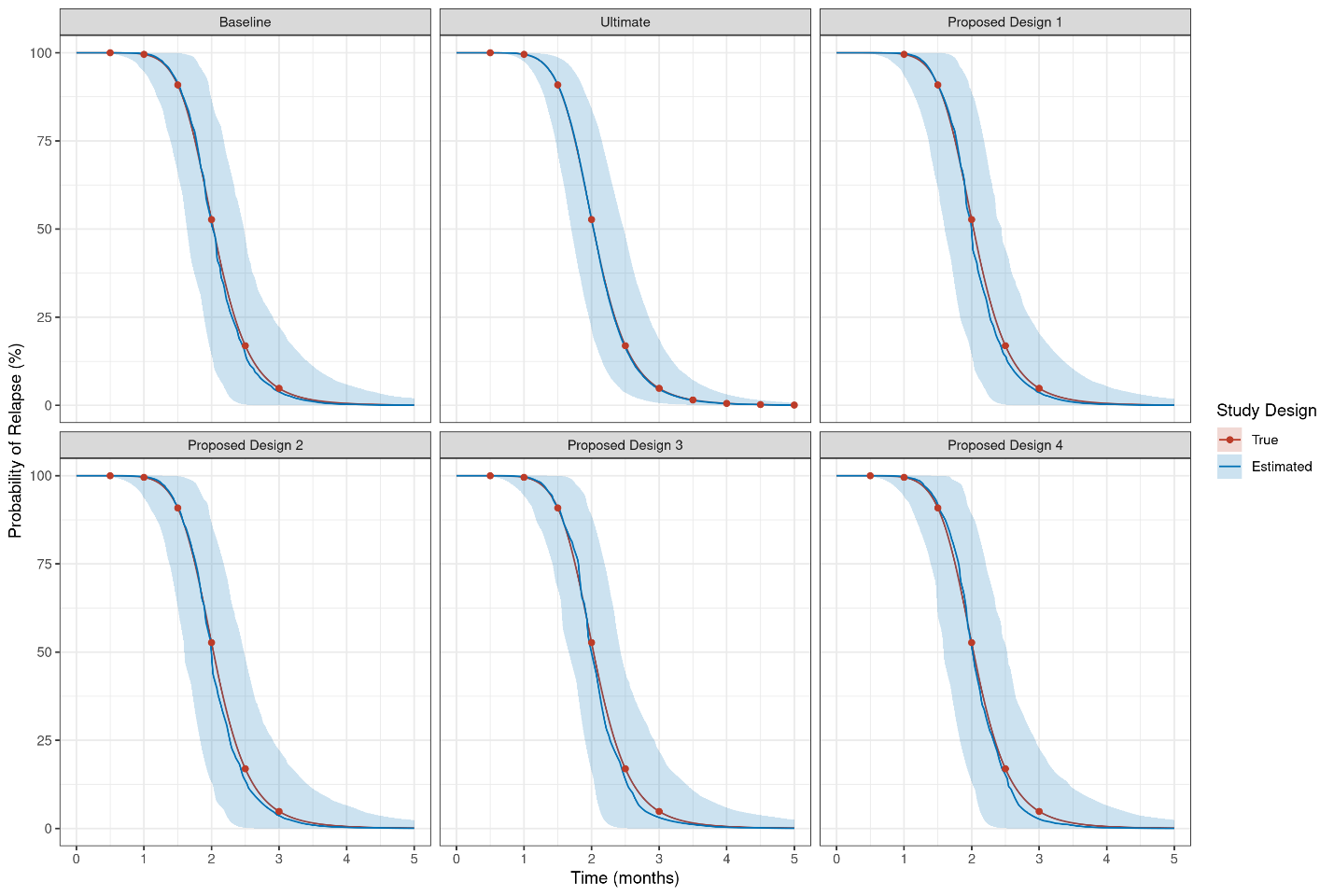
 **SFIG 11.** Relapse versus time profile for simulations of Regimen 7 by Design for simulation round 1. Blue lines and areas represent median and 90% confidence intervals for simulations. Red lines and dots are the simulation input.

**
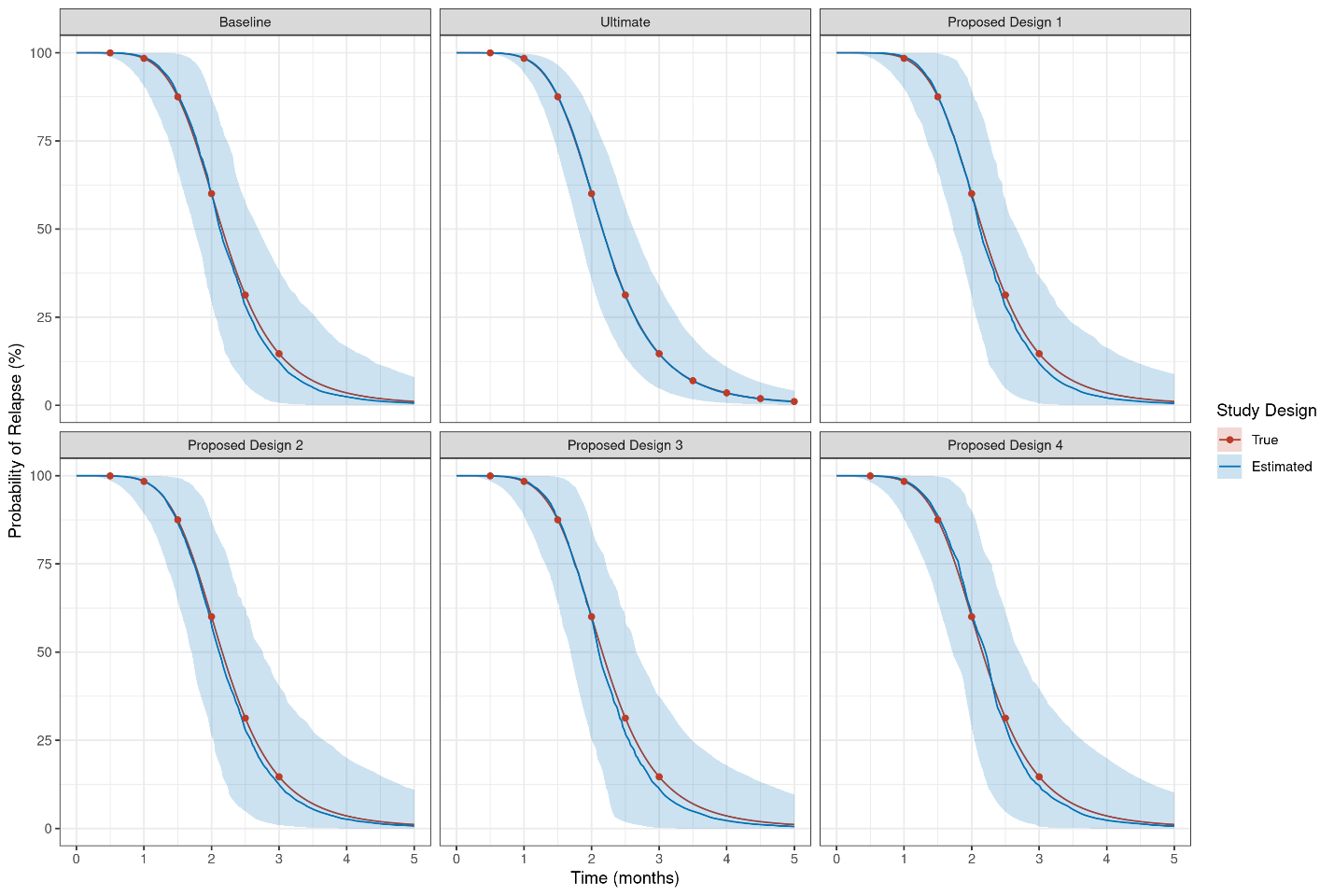
 SFIG 12.** Relapse versus time profile for simulations of Regimen 8 by Design for simulation round 1. Blue lines and areas represent median and 90% confidence intervals for simulations. Red lines and dots are the simulation input.


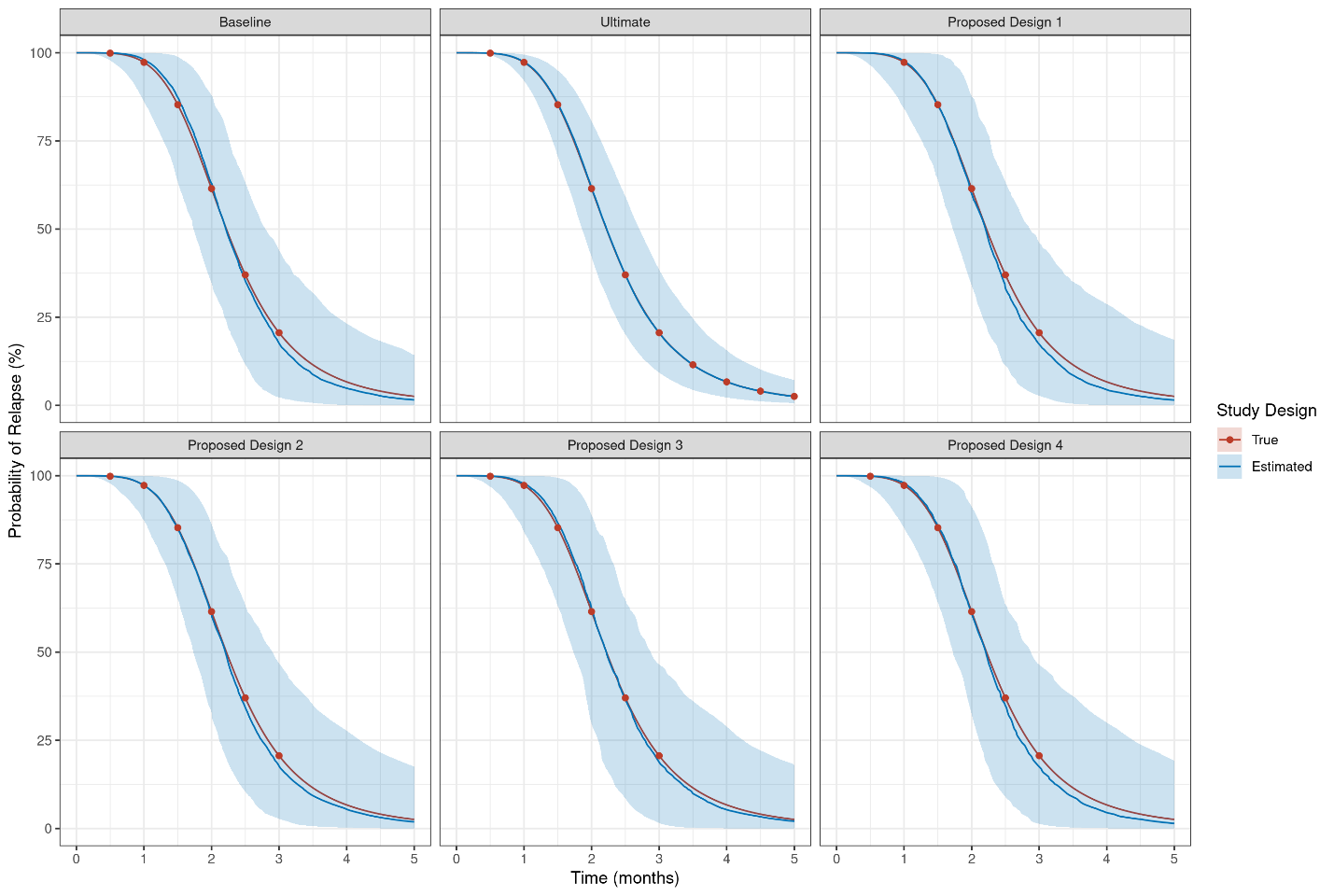
 **SFIG 13.** Relapse versus time profile for simulations of Regimen 9 by Design for simulation round 1. Blue lines and areas represent median and 90% confidence intervals for simulations. Red lines and dots are the simulation input.


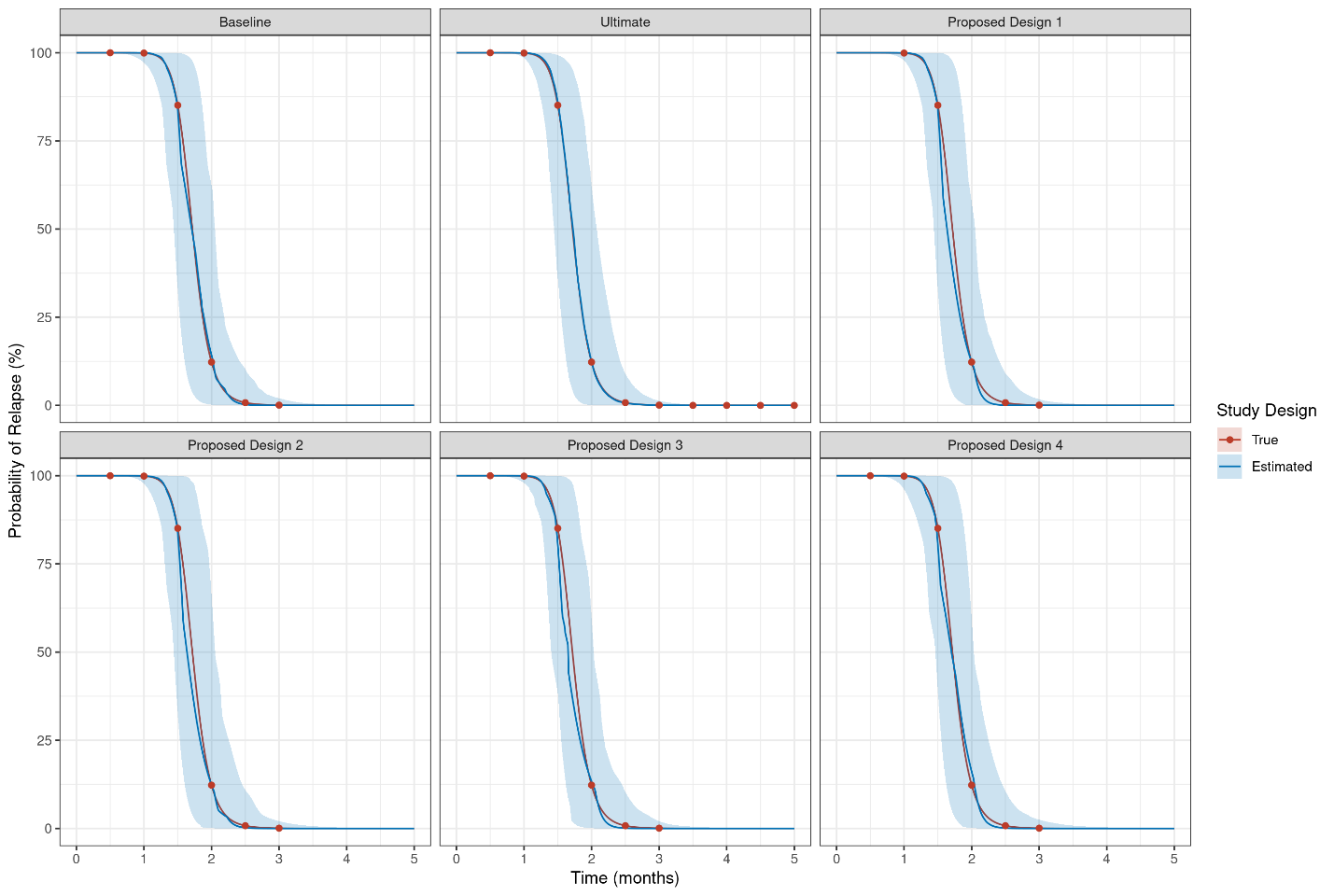
 **SFIG 14.** Relapse versus time profile for simulations of Regimen 10 by Design for simulation round 1. Blue lines and areas represent median and 90% confidence intervals for simulations. Red lines and dots are the simulation input.


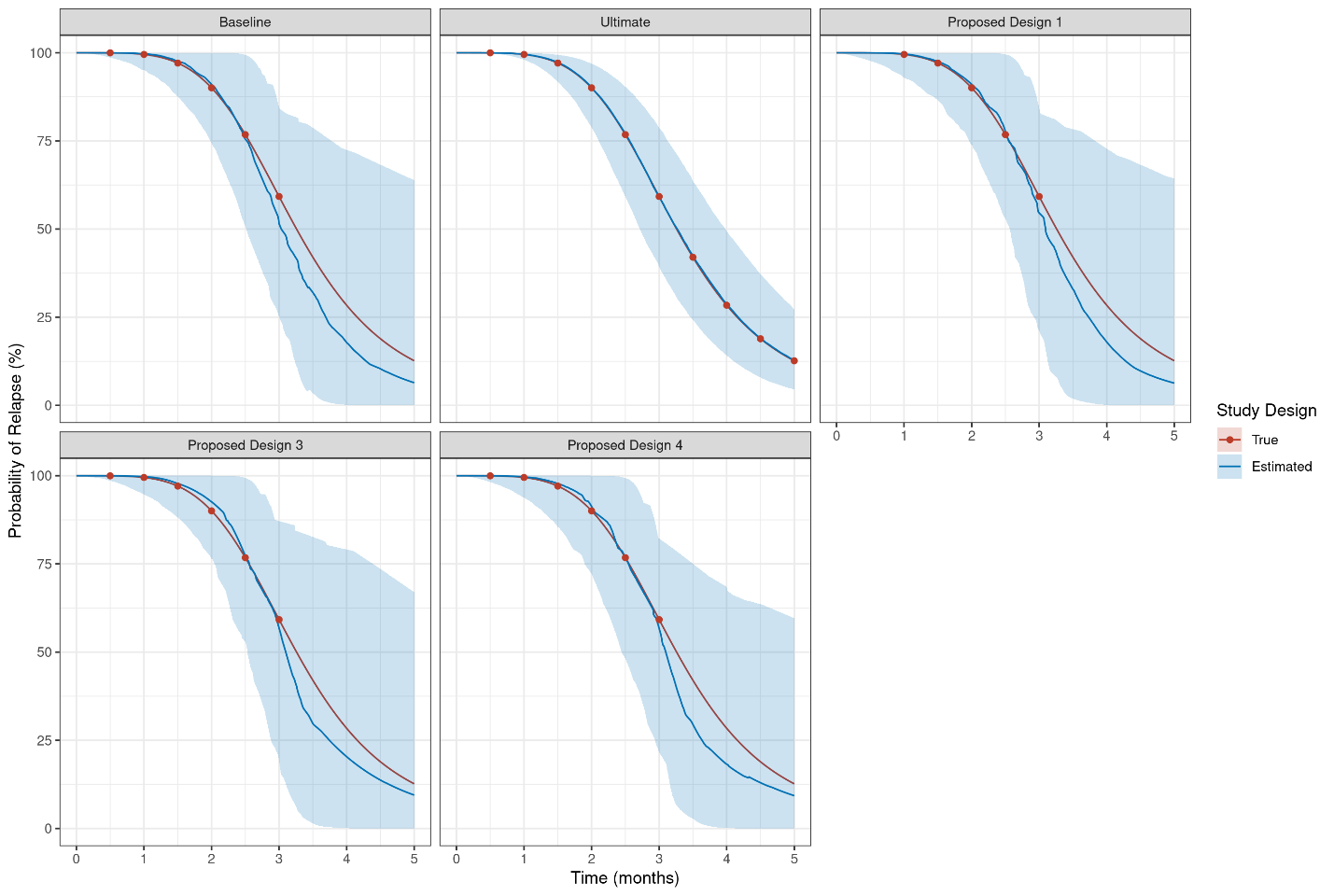
 **SFIG 15.** Relapse versus time profile for simulations of Regimen 11 by Design for simulation round 1. Blue lines and areas represent median and 90% confidence intervals for simulations. Red lines and dots are the simulation input.

**
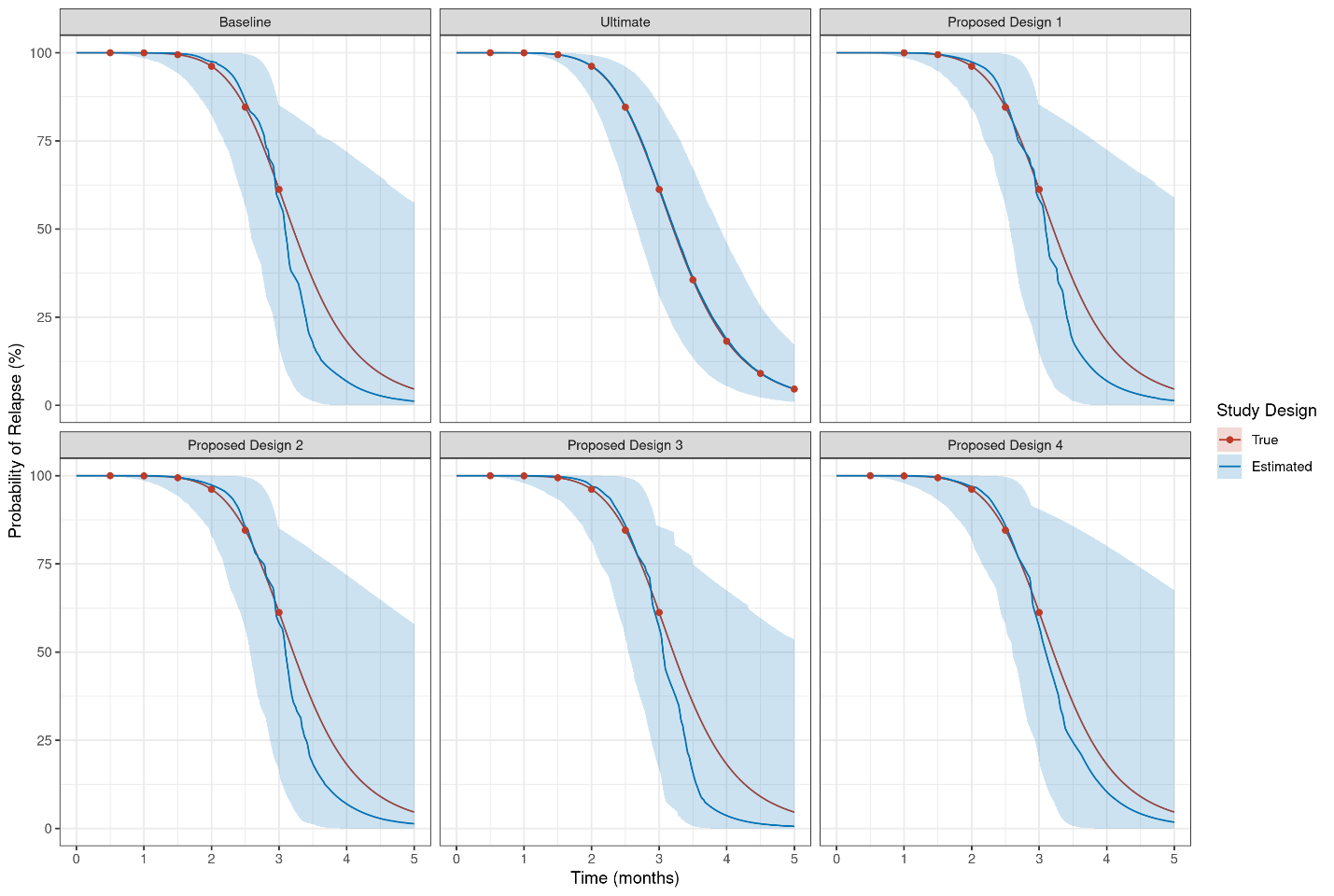
 SFIG 16.** Relapse versus time profile for simulations of Regimen 12 by Design for simulation round 1. Blue lines and areas represent median and 90% confidence intervals for simulations. Red lines and dots are the simulation input.
